# The precedence effect in spatial hearing manifests in cortical neural population responses

**DOI:** 10.1186/s12915-022-01228-z

**Published:** 2022-02-16

**Authors:** Kongyan Li, Ryszard Auksztulewicz, Chloe H. K. Chan, Ambika Prasad Mishra, Jan W. H. Schnupp

**Affiliations:** 1 Department of Biomedical Sciences and Department of Neuroscience, City University of Hong Kong, Hong Kong, SAR China; 2 City University of Hong Kong Shenzhen Research Institute, Shenzhen, China

**Keywords:** Precedence effect, Onset dominance, Rat, Temporal weighting function, Neural decoding, Auditory cortex

## Abstract

**Background:**

To localize sound sources accurately in a reverberant environment, human binaural hearing strongly favors analyzing the initial wave front of sounds. Behavioral studies of this “precedence effect” have so far largely been confined to human subjects, limiting the scope of complementary physiological approaches. Similarly, physiological studies have mostly looked at neural responses in the inferior colliculus, the main relay point between the inner ear and the auditory cortex, or used modeling of cochlear auditory transduction in an attempt to identify likely underlying mechanisms. Studies capable of providing a direct comparison of neural coding and behavioral measures of sound localization under the precedence effect are lacking.

**Results:**

We adapted a “temporal weighting function” paradigm previously developed to quantify the precedence effect in human for use in laboratory rats. The animals learned to lateralize click trains in which each click in the train had a different interaural time difference. Computing the “perceptual weight” of each click in the train revealed a strong onset bias, very similar to that reported for humans. Follow-on electrocorticographic recording experiments revealed that onset weighting of interaural time differences is a robust feature of the cortical population response, but interestingly, it often fails to manifest at individual cortical recording sites.

**Conclusion:**

While previous studies suggested that the precedence effect may be caused by early processing mechanisms in the cochlea or inhibitory circuitry in the brainstem and midbrain, our results indicate that the precedence effect is not fully developed at the level of individual recording sites in the auditory cortex, but robust and consistent precedence effects are observable only in the auditory cortex at the level of cortical population responses. This indicates that the precedence effect emerges at later cortical processing stages and is a significantly “higher order” feature than has hitherto been assumed.

## Background

Having two healthy ears can bring considerable advantages, enabling us to use binaural cues to localize sound sources and to separate sound sources in a cluttered acoustic environment. However, sound localization is often rendered more complicated by the fact that every hard surface reflects sound waves, acting like a “sound mirror” and creating a mirrored sound source which interferes with the localization of the original source. Interestingly, we are usually unaware of the powerfully distorting effect such reverberation has on the sound waves that arrive at our eardrums. Our auditory pathways appear to be equipped with powerful “echo suppression” mechanisms, but their function and their physiological basis remain very poorly understood. One important part of this echo suppression, which has been studied in some detail, is the so-called “precedence effect.” This refers to the phenomenon that the perception of sound source direction puts great emphasis on the binaural cue values experienced during the first few milliseconds of a new sound burst. This reliance on sound onset cues is thought to reduce the confounds that can arise when the binaural cue values of later parts of the sound are distorted by interference from reverberant sound (for a review, see [1]).

The precedence effect has been recognized as a psychophysical phenomenon in humans for over 100 years (for a history, see [2]), and behavioral studies on rats [3] and cats [4] indicate that it may be a common feature of mammalian hearing. Previous animal work [3, 4] tended to use brief individual stimulus pulse-echo pairs delivered in the free field, which is fine for investigating whether or not there is a precedence effect, but it does not permit quantification of the relative weighting that the precedence effect might give to various elements of a stimulus composed of a rapid series of consecutive pulses. Understanding the perceptual weighting in pulse train stimuli has considerable potential ecological and translational importance. Firstly, because most animal vocalization sounds, including human vowels, are effectively (band-pass filtered) pulse trains. Secondly, because cochlear implant (CI) neuroprosthetic devices, which are used increasingly to treat profound to severe hearing loss, will typically encode auditory stimuli as rapid trains of electrical pulses delivered to auditory nerve fibers. Being able to measure the relative perceptual weight that the brain assigns to each pulse in a pulse train as it forms sound source location judgments is therefore likely to become important in the development of better sound processing algorithms for future assistive or neuroprosthetic devices.

One elegant way to perform such a quantification was introduced by Stecker and Hafter [5], who used brief binaural click trains delivered over headphones to measure so-called temporal weighting functions (TWFs) psychoacoustically. Each pulse in the train carries its own binaural cue parameters (interaural time or level differences, ITDs or ILDs), but pulses are delivered at a rate high enough for clicks to fuse perceptually into a single perceived sound. Subjects are asked to indicate the perceived source direction of the click train, and a statistical analysis is used to calculate the relative influence of each click in the train on the overall perceived source direction. Studies using this paradigm on normally hearing human subjects [6–9] have consistently shown a strong onset dominance to click trains, which is more pronounced for higher click rates than for lower click rates.

From a translational perspective, it would be of interest to extend this type of approach to hearing-impaired patients as well as to animal models suitable for the study of hearing loss and treatments. It is well known that patients with severe hearing loss often have great difficulty processing binaural cues, even if they are fitted with bilateral CIs [10]. The processing of ITDs appears to be particularly severely affected in such patients, even more so than that of ILDs, and in this study, we shall focus on ITDs as they are the type of binaural cue for which deficits, and therefore the need for improvement, are greatest. Some recent studies have raised the intriguing possibility that changes to the current standard algorithms for computing CI stimulus pulses might enhance the ITD sensitivity of bilateral CI patients. For example, introducing temporal jitter into the interval between stimulus pulses may enhance the sensitivity to ITDs in bilateral CI users [11] as well as normally hearing listeners [12]. Meanwhile, Litovsky [13] reported that bilateral CI users and normally hearing children may not exhibit a precedence effect, which raises questions about how stimulation paradigms and neurobiological development interact to further result in effective spatial auditory perception. We will need a better understanding of the technical factors and biological mechanisms that determine binaural listening performance to improve patient outcomes. The development of a suitable animal model that lends itself to combined behavioral and physiological approaches is going to be an important step in this endeavor.

Our recent papers [14] demonstrated that Wistar rats have similar sensitivity to both onset and ongoing envelope ITDs as humans when tested in a sound lateralization task with pulsatile stimuli, and intriguingly, rats fitted with bilateral CIs can exhibit essentially normal behavioral ITD sensitivity even after prolonged neonatal hearing loss [15]. This is in stark contrast to human CI patients, who, as already mentioned, often have very poor ITD sensitivity [10]. Rats may thus be a highly valuable model for the study of binaural hearing and the development of better auditory prosthetic strategies. To evaluate their potential usefulness, it would be helpful to know if rats also exhibit similar temporal weighting as human subjects do. In this study, we therefore sought to answer the following three questions: (1) whether it is possible to adapt the TWF approach developed by Brown and Stecker [6] for behavior testing in rats, (2) whether behaviorally measured ITD TWFs for rats resemble those reported for humans, with most or all of the perceptual weight vested in the first click of a train, and (3) whether or how electrophysiological responses recorded from rat auditory cortex (AC) reflect the behavioral weighting.

## Results

### Behavioral data revealed profound onset dominance

For the behavioral experiment, female Wistar rats were trained to perform a two-alternative forced-choice (2-AFC) near-field lateralization task very similar to that described in [14]. The animals initiated a trial by licking a center spout, were presented with an acoustic stimulus over tube phones, and then responded by licking response spouts on the left or right. Correct responses were positively reinforced with drinking water; incorrect responses triggered a short timeout. (See the “Behavioral training task” section for further details.) The rats were trained twice daily, 5 days a week. Usually, the rats would perform ~ 160 trials on average in each 20-min training session, but the number could vary from just below 100 to well over 200. The rats were initially trained with a 200-ms-long, 300-Hz binaural click train including both ITD (± 0.136 ms) and ILD (± 6 dB) cues for 13 to 17 training sessions until they reached the 80% correct criterion in at least two sessions. Our convention here is to use negative values to indicate binaural cues that favor the left ear. These binaural cue values were deliberately chosen to be large compared to the animals physiological range, to make the stimuli easy to discriminate and allow the animals to work on learning the procedure without having to struggle with difficult sensory discriminations. In particular, acoustic measurements on rats [16] indicate that ITDs of ~ 0.13 ms are at the upper limit of the animals’ physiological range, and would therefore be associated with source directions near the intra-aural axis (± 90° azimuth). Upon reaching high levels of performance (> 80% correct), the rats then progressed to “ITD only training”, which kept the ± 0.136 ms ITD cue, but set ILD to 0 dB. The removal of the ILD cue had little effect on performance, so the rats required only 2 to 3 sessions to reach the criterion of > 80% correct in two sessions. They then progressed to multiple “ITD value training” meaning that, at each trial, stimulus ITDs were randomly chosen from the set ±[0.1587, 0.136, 0.0907, 0.068, 0.0454, 0.0227] ms. This introduced many new stimuli with smaller ITDs, closer to threshold, and the rats needed between 18 and 21 training sessions before reaching the respective criterion for this training phase (75% correct in two sessions). We initially trained 5 rats with this protocol, four of which reached the required high performance with ITD-only stimuli after about 2 weeks of training. The one rat which failed to achieve the performance criterion for progression after 2 weeks of training was excluded from the rest of the study.

**Fig. 1:**
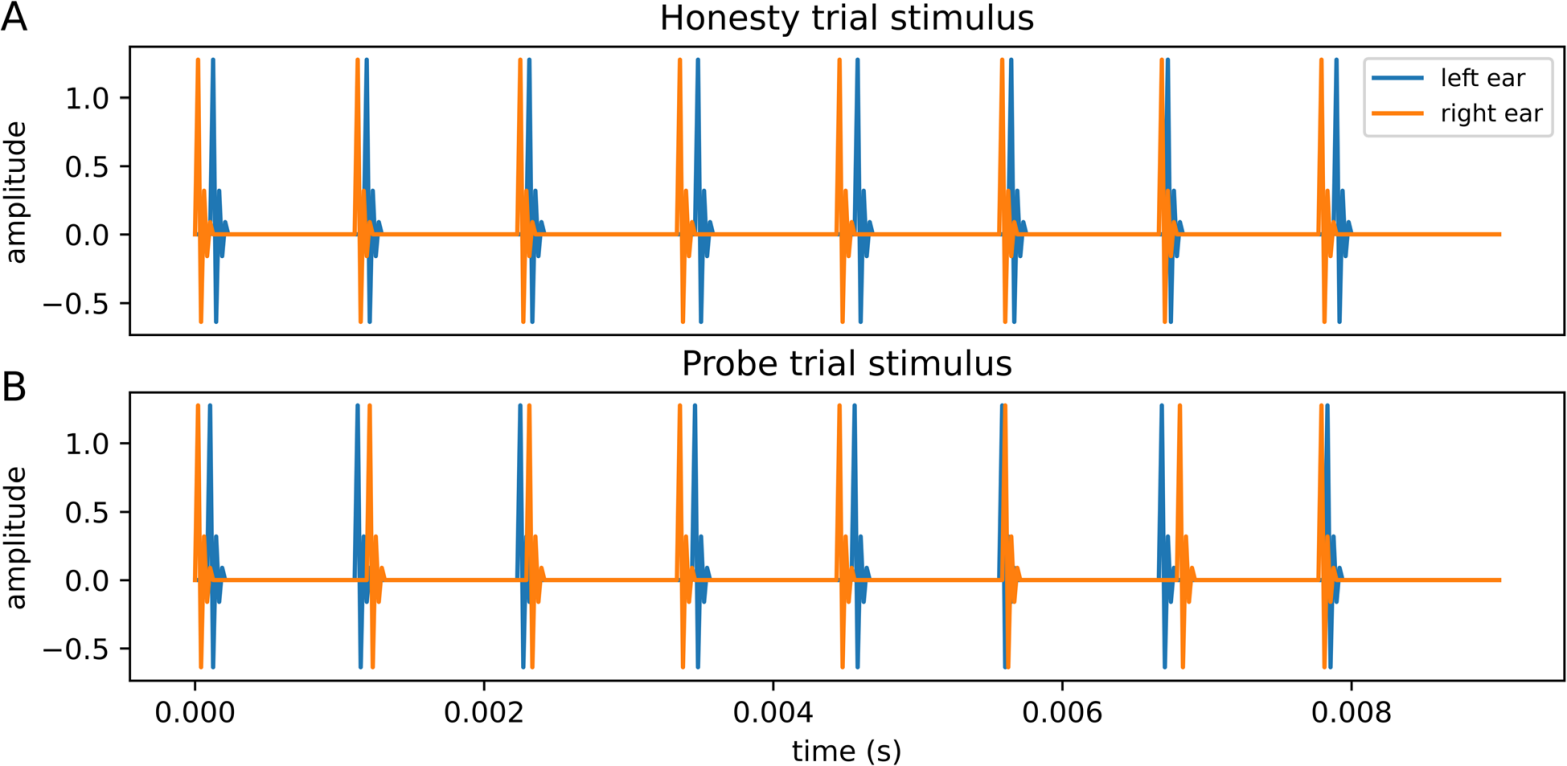
Example waveforms of the acoustic stimuli used in the behavioral experiments. **A** Example of an honesty trial stimulus at 900 Hz with 0.042 ms jitter and + 0.083 ms offset (+: right ear leading, −: left ear leading). In honesty trials, all ITDs point in the same direction (in this example the right ear is always leading, even if the size of the ITD can vary between + 0.042 and + 0.125 ms). There is no ambiguity, and the rat will only be rewarded for responding on the appropriate side. **B** Example of a probe trial stimulus at 900 Hz with 0.125 ms jitter and 0 ms offset. The ITD for each binaural pulse was randomly chosen from the range of − 0.125 ms to + 0.125 ms. Since there was no a priori objectively correct response to probe trials, rats were rewarded in those trials irrespective of which side they responded on

After completing the multiple ITD values training, the rats were introduced to stimuli designed to measure their TWFs (see section “Acoustic stimuli for behavioral TWF measurement” below for further details). Briefly, our TWF stimuli consisted of a brief train of eight binaural pulses presented at a rate of 20, 50, 300, or 900 Hz, with the ITD for each of the pulses drawn independently and uniformly from the range of ± 0.125 ms. We generated two types of such TWF stimuli: In stimuli for “honesty trials” (see Fig. 1A), the ITDs for all 8 pulses were either positive (right ear leading) or negative (left ear leading), and the rat had to respond on the appropriate side to receive a reward. In contrast, in “probe trial” stimuli (Fig. 1B), the ITDs were unconstrained, there is not a priori correct lateralization, and the animals were free to choose to respond as they pleased and were rewarded regardless. The final training stage before formal testing began involved only honesty trials. Once the rats’ performance exceeded 80% correct in two or more sessions, testing commenced with TWF stimulus sessions containing both “honesty” and “probe” trial stimuli presented randomly interleaved in a 2:1 ratio. For the 50-Hz and 300-Hz pulse rates, only 2 honesty-trial-only training sessions were needed for all the rats before testing could begin, while for the 20-Hz and 900-Hz pulse rates, 4 and 6 training sessions respectively were required before the final behavioral testing stage.

In the final, psychoacoustic testing stage, we collected data over a total of 35 sessions for rat 1801, 34 sessions for rat 1802, 33 sessions for rat 1803, and 33 sessions for rat 1805. The numbers of probe and honesty trials and the correct response rate in honesty trials in each condition for each rat are summarized in Table 1. Rat 1802 was the best performer in honesty trials for all pulse rates. The correct rate in honesty trials and the number of training sessions needed in the honesty training stage suggest that the task difficulty was similar across the four tested click rates. Note that, even at the most difficult, 900 Hz click rate, all rats had more than 80% correct responses in honesty trials.

**Table 1:** A summary of the data collected in the final “honesty + probe” testing stage

Condition	Animal	Probe trials	Honesty trials	Correct responses in honesty trials	Correct response rate in honesty trials
20 Hz	1801	558	1095	879	80.27%
	1802	517	1077	984	91.36%
	1803	564	1152	983	85.33%
	1805	626	1163	946	81.34%
50 Hz	1801	458	888	759	85.47%
	1802	495	923	844	91.44%
	1803	469	898	763	84.97%
	1805	457	943	765	81.12%
300 Hz	1801	449	928	784	84.48%
	1802	525	986	881	89.35%
	1803	423	816	675	82.72%
	1805	422	814	652	80.10%
900 Hz	1801	399	796	643	80.78%
	1802	413	868	800	92.17%
	1803	388	798	657	82.33%
	1805	410	882	707	80.16%
Total		7573	15027	12722	84.66%

**Fig. 2:**
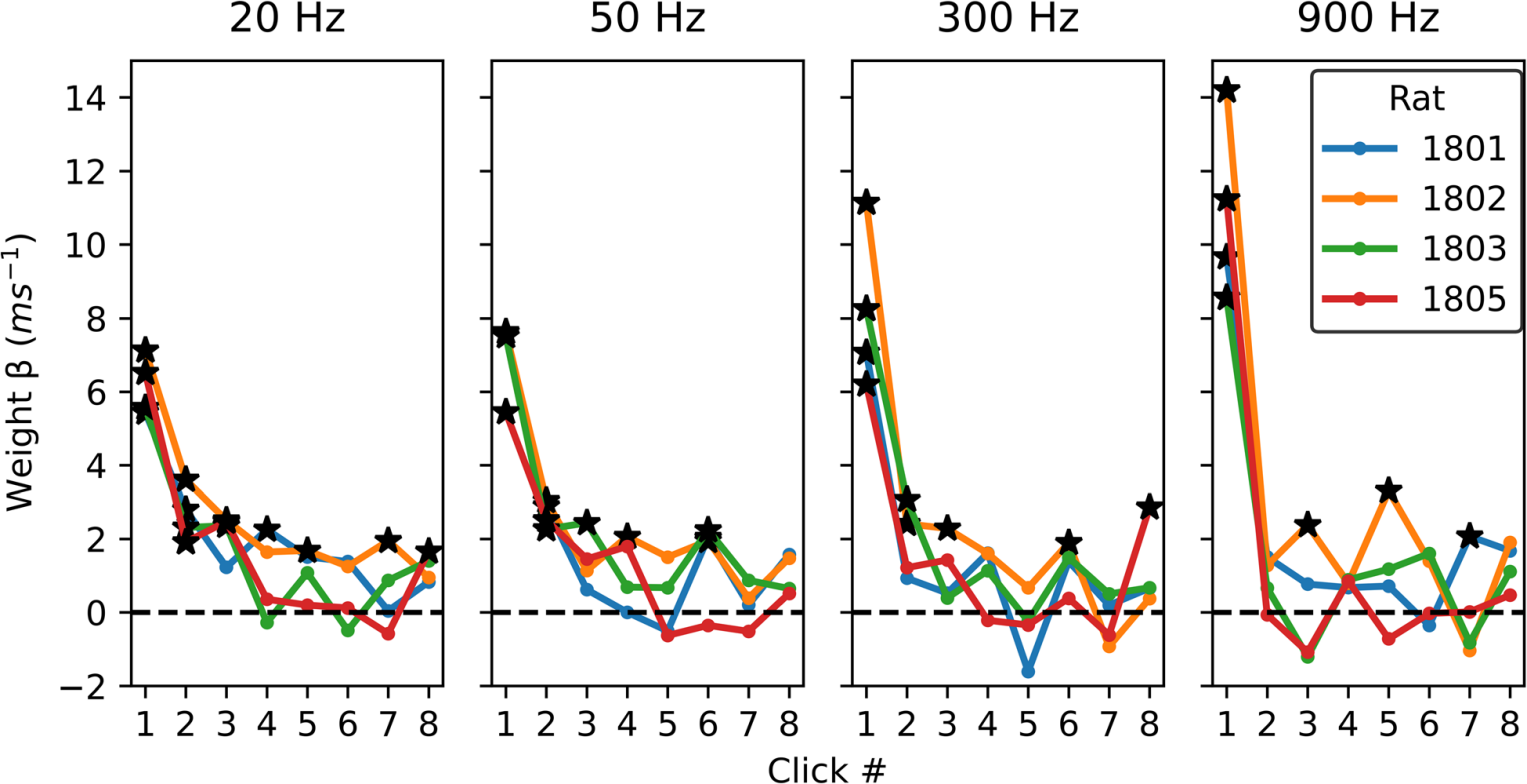
ITD Temporal weighting functions for the four rats used in the behavior experiments, at the click rates shown above each panel. The *x*-axes show the individual clicks in the 8-click train. The *y*-axes show the “temporal weight” of the corresponding click as calculated via Probit regression. Weights which were individually statistically greater than zero are based on the *p* values obtained in the Probit regression (*p* < 0.01) and are marked with asterisks. Strong onset dominance was seen at all pulse rates for all rats. The weights of the clicks following the first click increased as the click rate decreased

An analysis of the probe trials obtained during these testing sessions using Probit regression revealed a profound and consistent onset dominance (“precedence effect”) across all animals and all click rates. The results were also remarkably consistent across all animals tested, as can be appreciated in Fig. 2, which shows the TWFs, computed as Probit coefficients of each for the eight clicks in our click trains (see section “Behavioral data analysis”), for each of the four animals and at each of the four pulse rates tested. The figure allows us to appreciate the high consistency of the behavior results across all animals in our cohort. The first and second pulse weights were significantly above zero in all 4 animals for click rates of 50 Hz or less, but for higher click rates of 300 and 900 Hz, significantly non-zero weights beyond the first pulse are only observed sporadically.

**Fig. 3:**
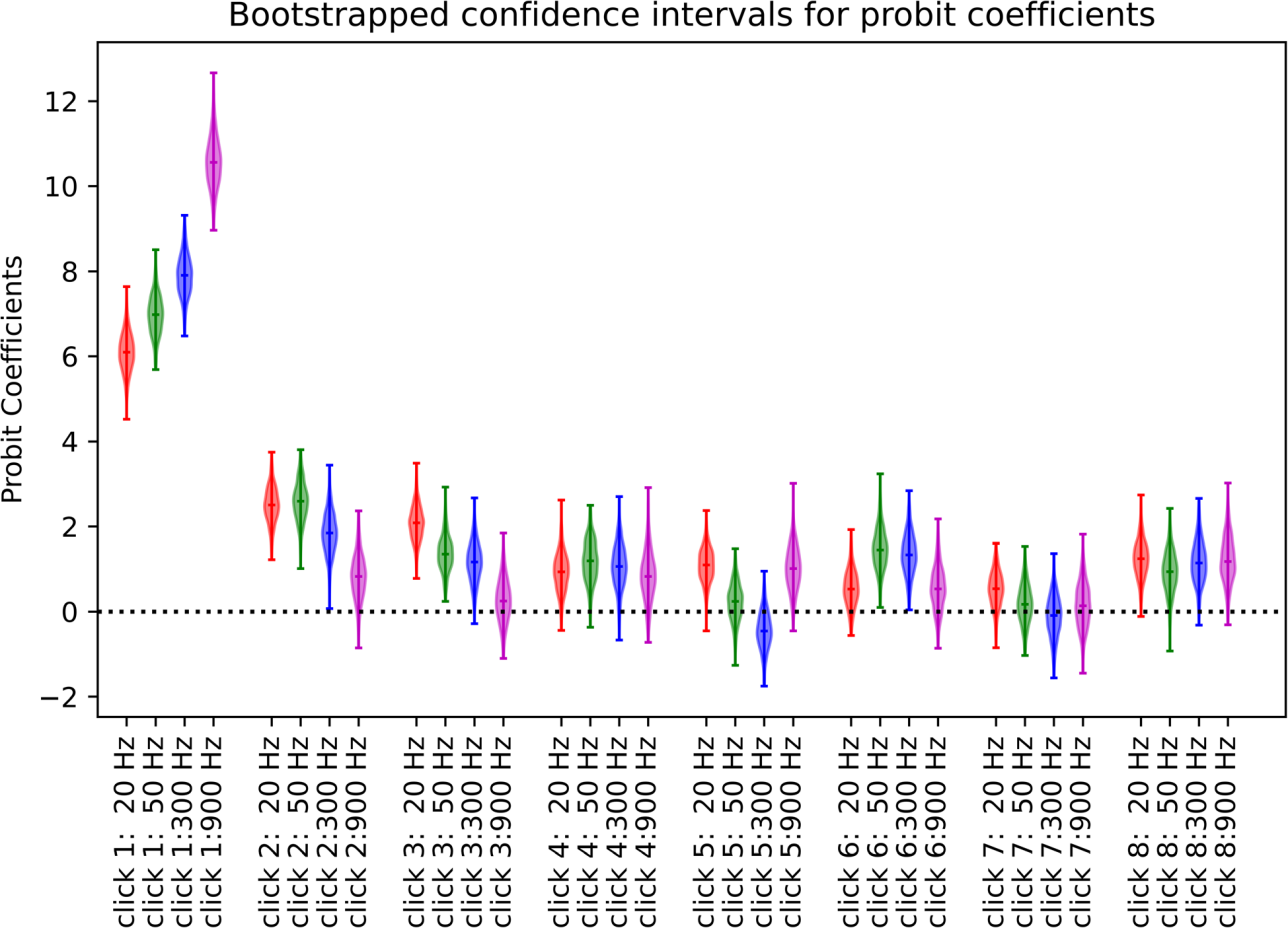
Distribution of Temporal Weights (Probit Coefficients) obtained by resampling the behavioral data. The colored lines show the range and the median values of the coefficient distributions obtained over 1000 bootstrap trials. The spindle shapes display the kernel densities of the corresponding distribution: their widths reflect the proportion of bootstrap samples over a given narrow *y*-range of coefficients in much the same way as the height of a histogram would

It is also noticeable that faster pulse rates produced the strongest weighting for the first pulse, suggesting greater onset weighting at higher pulse rates. This is entirely consistent with previous reports of similar trends in human listeners [8]. In order to estimate whether such differences in weights are statistically significant, we computed bootstrap confidence intervals for the Probit regression weights using classic N-out-of-N resampling. Behavioral data were pooled across all 4 animals, and the set of N trials for each pulse rate was randomly resampled with replacement to generate a bootstrap sample of size N, for which Probit coefficients were computed in the same way as for the original data set. This random resampling was repeated 1000 times to generate a bootstrap distribution of temporal weights. The resultant bootstrap distributions for the temporal weights are represented graphically as violin plots in Fig. 3. The full extent to these distributions can be interpreted as 99.9% confidence intervals for the true temporal weights. Note that these do not overlap for click 1 at 20 Hz vs click 1 at 900 Hz, indicating that the observed trend for initial weights to increase with pulse rate is statistically robust. Also note that the bootstrap distributions are entirely above zero at 20 Hz for pulses 1, 2 and 3, while at 900 Hz only the first pulse had a weight that can be considered significantly greater than zero. This suggests that higher pulse rates not only produce larger initial weights, but also a more rapid decay of subsequent weights. How complete is this decay? To answer this question, we can examine the bootstrap distributions for the later pulses, for example, from click 4 onward. Note that, although almost all (14 out of 16) of the bootstrap distributions for pulses 4 to 8 straddle zero, nevertheless, the great majority of them (14 out of 16) have medians greater than zero. Such a large proportion of above zero medians would be surprising if the later pulses contributed absolutely nothing to the rats’ lateralization percept. For comparison, the binomial probability of observing 14 or more “heads” in 16 flips of a fair coin would be only 0.0021. Thus, the contributions of the later pulses to the lateralization percept are clearly very small, much smaller than that of the initial pulse and so small as to be hard to measure accurately, but they nevertheless appear to be, on average, slightly greater than zero.

In summary, the behavioral results shown in Figs. 2 and 3 demonstrate that rats temporally weight ITD cues in a manner that is very similar to the temporal weightings previously described for human listeners by Stecker and colleagues [5, 8, 17]. All four rats in our cohort showed a very strong and highly consistent onset weighting at all tested pulse rates, and the strength of onset weighting increased significantly at higher pulse rates.

### Channel-wise regression shows only sporadic precedence effects in electrocorticographic (ECoG) signals from the auditory cortex

Having demonstrated that rats exhibit strong temporal onset weighting in their behavioral lateralization of acoustic stimuli which closely resembles that seen in humans, we wanted to test whether neural responses in auditory cortex showed a similarly strong onset weighting, as might be expected if cortical ITD tuning provides a simple and direct neural correlate of the sensations the rats report in their behavior. However, identifying the neurophysiological correlates of TWFs is more challenging than measuring TWFs behaviorally, since neurons in the auditory pathway do not give binary “left” or “right” responses, but instead have ITD tuning curves which can differ greatly from one neuron to the next. Furthermore, these tuning curves may map ITD values to neural firing rates in a non-monotonic manner [18]. Field potentials recorded with ECoG electrodes will reflect a superposition of many such diverse tuning curves. Measuring neural TWFs can therefore degenerate into an under-constrained problem, where one seeks to understand the mapping of a relatively large number of continuous-valued stimulus parameters (the ITDs of each pulse in the train) onto a very noisy continuous-valued output variable (electrical signals reflecting neural firing rates) through a set of tuning curves of unknown shape. In an attempt to make the problem more tractable, we reduced the complexity of the stimuli compared to those used in the behavioral experiment by reducing the number of pulses in the train from 8 to only 4, and by constraining each pulse so that it could take only one of two possible ITD values, either − 0.164 ms or + 0.164 ms. These ITD values are close to, and slightly larger than, the maximal ecological ITD values of ± 0.13 ms measured acoustically in rats [16]. By constraining each stimulus pulse train to have only four pulses, and each pulse constrained to take one of only two possible values (corresponding to “far left” or “far right”), we reduced the set of all possible stimulus pulse trains to only 16, and we presented each of these 16 possible stimuli 40 times, in pseudo-random order, recording 640 responses in total at each recording site. We refer to this reduced-complexity set of stimuli as “sparse” TWF stimuli to distinguish it from the richer set of stimuli used in the behavior.

**Fig. 4:**
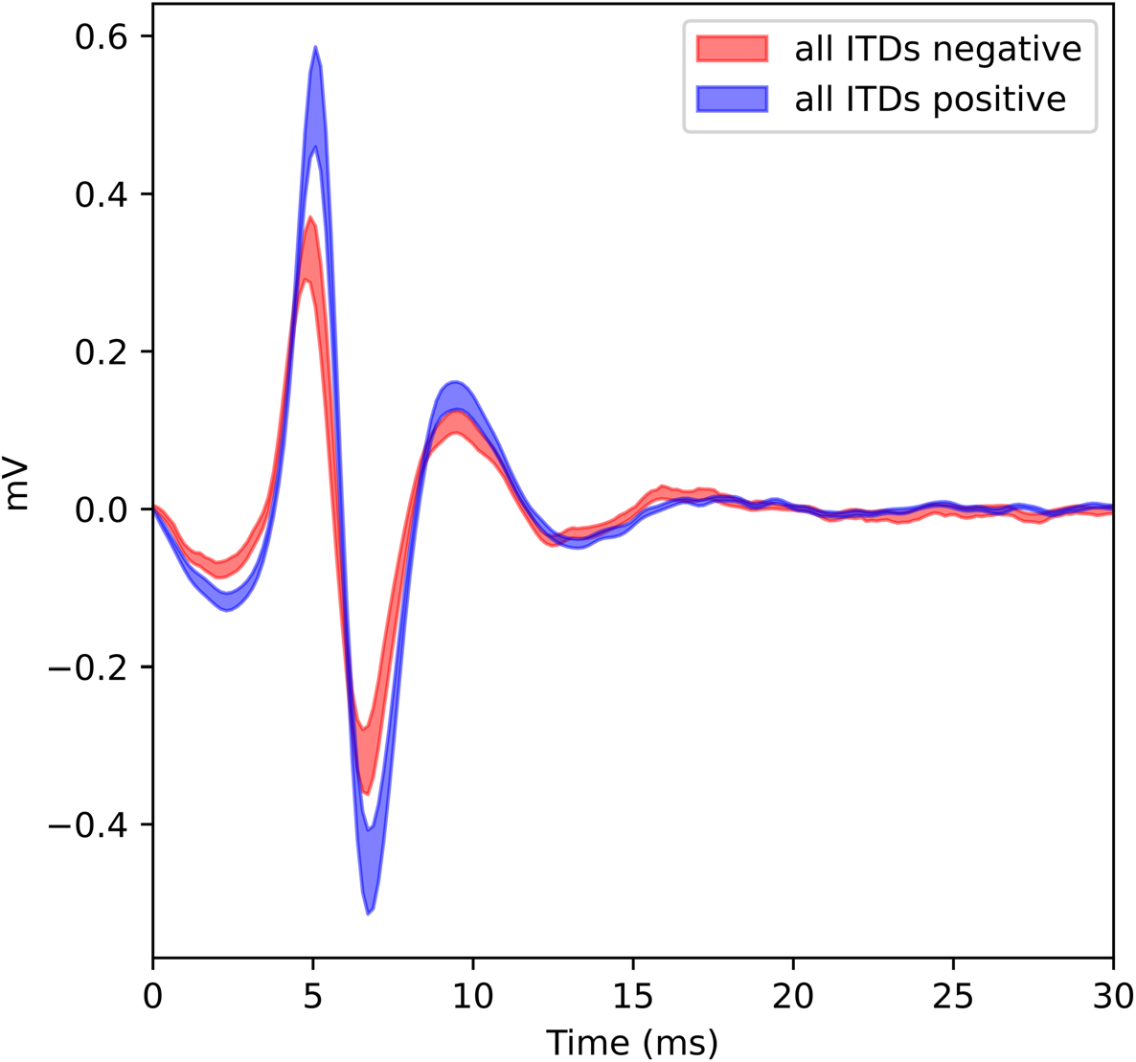
Examples of LFPs recorded at channel 29 from the left cortex of rat 1803. The stimulus were “sparse” ITD stimulus pulse trains (consisting of only four binaural pulses) at 300 Hz. The lines show mean ± standard error of the LFP responses recorded when either all four ITDs were + 0.164 ms (red line) or when all were − 0.164 ms (blue line). In this particular example, left ear leading ITDs gave the stronger response, even though this channel was recorded on the left side, contrary to the expectation that cortical neurons generally prefer sound source locations from the contralateral side. ECoG channels which defied that expectation were not uncommon in our dataset

In total, we recorded electrophysiological responses to this set of sparse TWF stimuli at 12 ECoG electrode placements: 4 placements from the right AC of each of the 4 trained animals in our cohort, and another 3 from the left AC of 3 of the 4 trained animals, plus another 5 recordings from the right AC of an additional five untrained animals which had not been exposed to the TWF stimuli prior to the electrophysiological experiments. At each electrode placement, we recorded responses to our sparse TWF stimuli at two pulse rates: 300 Hz and 900 Hz, yielding a total of 24 electrophysiology data sets. As described in the “Univariate analysis: channel-wise regression” section, in our univariate regression analysis, we aimed to quantify how well changes in LFP response amplitudes could be accounted for by a linear regression model in which each of the four ITDs of our “sparse” ITD stimulus set serve as regressors. This approach does of course rely on the assumption that LFP amplitudes recorded with our ECoG arrays do covary with stimulus ITD, at least to some extent and for some electrode channels. It is well documented that single units recorded in the auditory cortex with penetrating, high-impedance electrodes can be ITD tuned. However, one cannot necessarily take it for granted that ITD sensitivity is still observable in ECoG electrode signals recorded from the cortical surface. In Fig. 4, we therefore show example LFPs recorded from a single ECoG channel with two different stimuli, one where all four ITDs were − 0.164 ms, the other where all four ITDs were + 0.164 ms. It is readily apparent that the LFP amplitude is somewhat smaller for the − 0.164 ms ITD case. For this particular channel, that difference in LFP amplitude is highly statistically significant (*p* = 0.0026, rank sum test), but that channel was selected as an illustrative example to motivate the overall approach. Not all recording channels can be expected to show such a clear and large ITD dependence on response amplitude. The frequent ipsilateral ITD tuning is unexpected. Nevertheless, if we assume that the precedence effect is established early in the pathway, perhaps by cochlear mechanisms or inhibition in the brainstem and midbrain (we will revisit these notions in the discussion), then we would expect that the type of ITD tuning observed in Fig. 4 should be dominated by the first pulse in the train. Furthermore, this dominance ought to be manifest in a multiple linear regression that computes a set of regression parameters *β*_*n*_ to quantify how strongly RMS LFP amplitude depends on the ITD values of each of the four pulses in the stimulus (see section “Univariate analysis: channel-wise regression” for details).

**Fig. 5:**
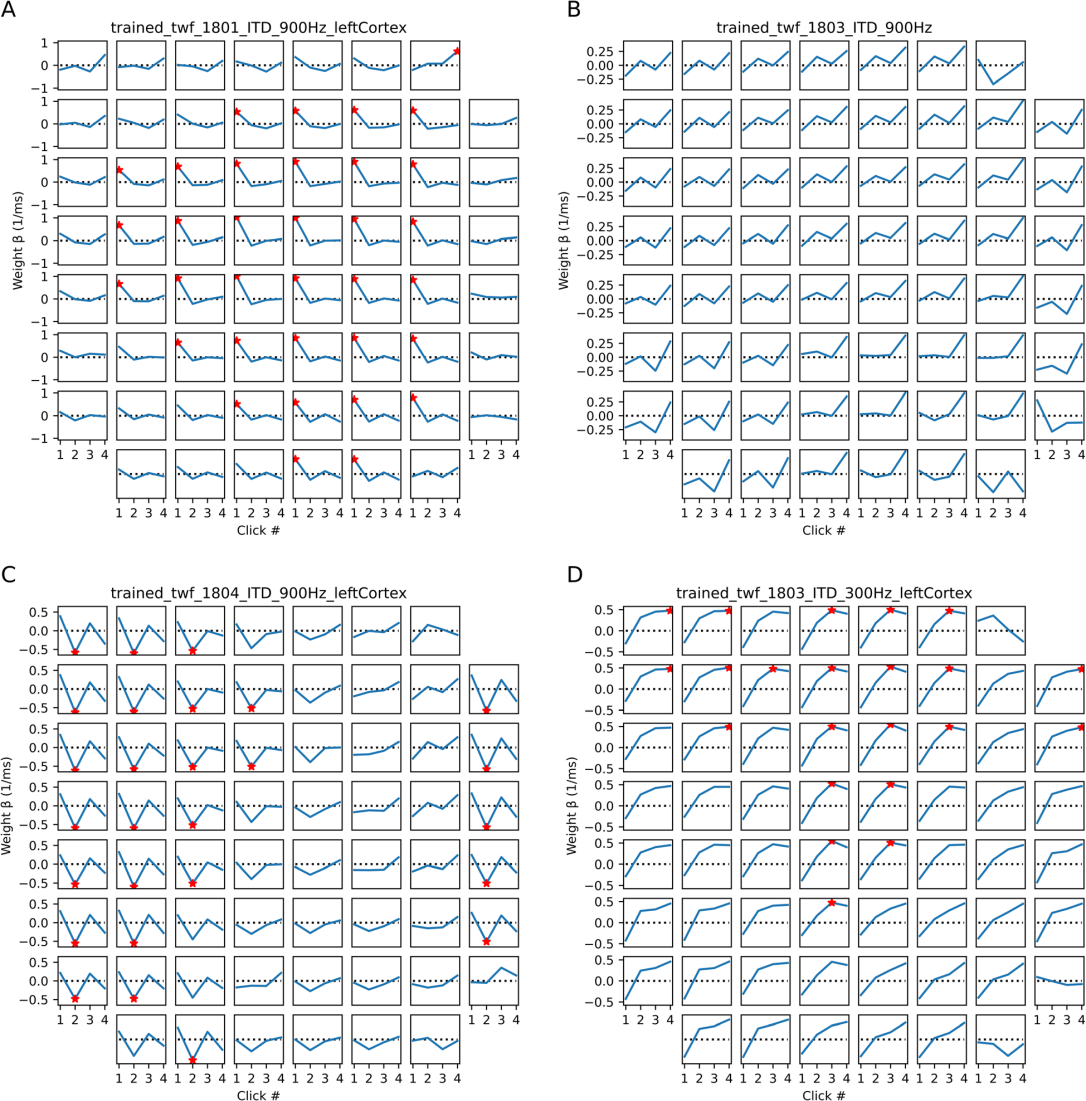
Examples of physiological TWFs obtained by univariate analysis of auditory cortex ECoG responses. In each subplot, the *x*-axis shows the individual click in the 4-click train, and the *y*-axis shows the beta value (“temporal weight”) from linear regression, in units of proportion change in normalized response per ms ITD, computed with ordinary least squares regression. Each subplot represents the physiological TWF obtained by analyzing the responses recorded at a different ECoG array position and pulse rate. The examples are chosen to be representative of the very diverse results obtained. Red asterisks (*) mark regression weights that were individually significantly different from zero at *p* < 0.05 (not corrected for multiple comparisons). Some physiological TWFs showed strong and significant onset weighting (e.g., most channels in **A**), but many others did not, and it was not uncommon for temporal weights other than the first to be the largest absolute significant value

However, in contrast to the behavioral experiments, which showed highly consistent and strong weighting of the first pulse, the results of the regression analysis of the ECoG results were highly variable, and not very consistent in which of the four pulses most strongly shaped the response. Overall, we observed at best only a weak trend for slightly larger weights for the ITD of the first pulse compared to later pulses in the train, and there was a great deal of heterogeneity in physiological TWFs from animal to animal and from recording site to recording site. Physiological TWF shapes could also vary quite strongly depending on whether the pulses were presented at 300 or 900 Hz. Only for a small subset of ECoG electrode placements did we observe a clear and statistically significant weighting of the first pulse in a majority of channels. An example is shown in Fig. 5A. For many other electrode placements, we saw a much more mixed picture, without a convincing or consistent tendency for the first pulse to carry the highest weight, as would be expected if neural firing rates consistently reflected the behavioral weighting. The examples in Fig. 5B–D illustrate the diversity of physiological TWFs obtained, the strong trend for TWFs to correlate among neighboring channels, and the fact that in these univariate physiological TWFs the largest absolute weights can also often occur on the 2nd, 3rd, or 4th, rather than the 1st pulse.

**Fig. 6:**
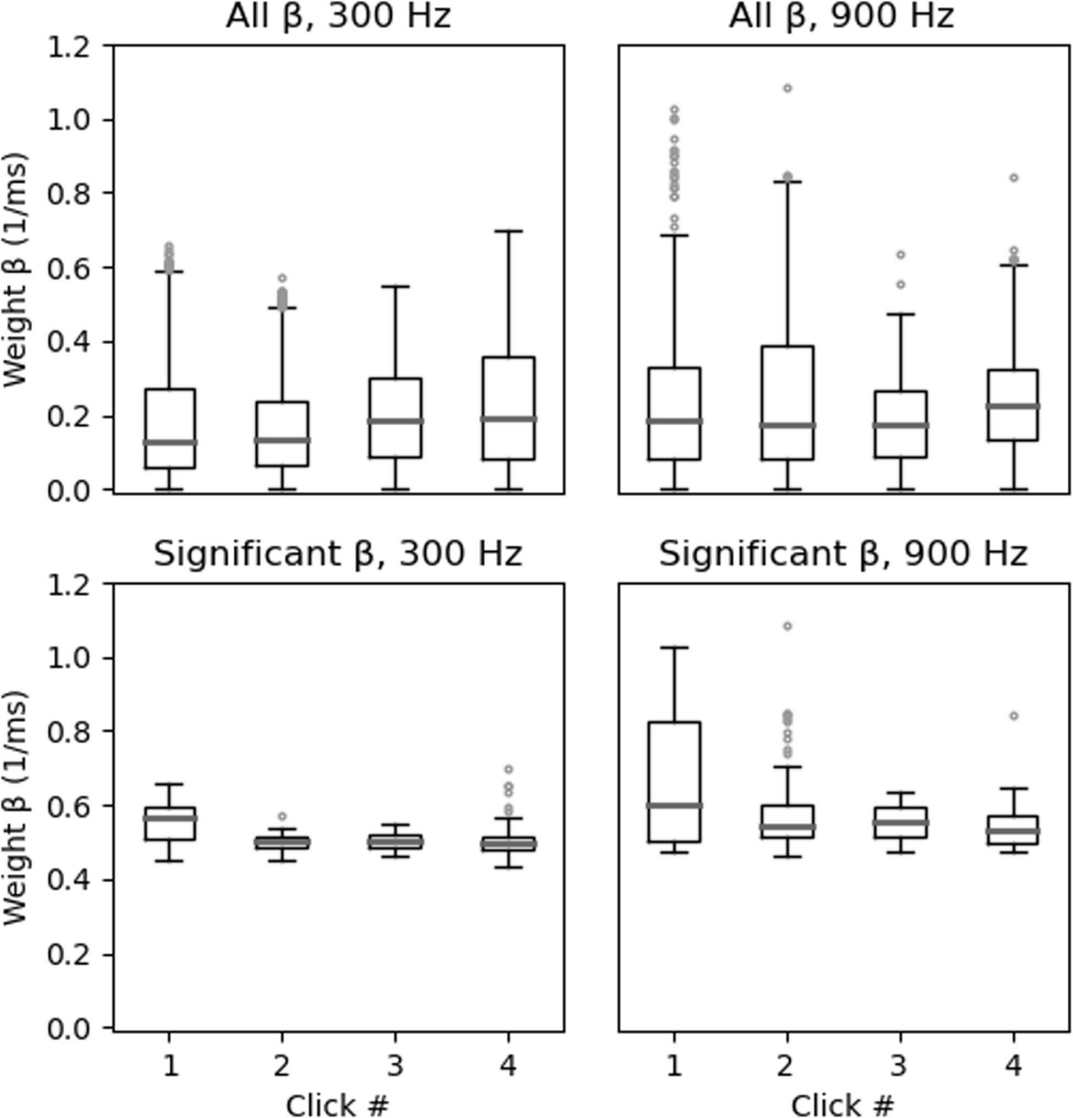
Boxplots showing the distributions of absolute weights at 300 Hz and 900 Hz pulse rates. The weights were pooled across all ECoG recordings from all nine rats. The *x*-axis indicates the individual click in the 4-pulse train, and the *y*-axis shows the absolute beta value (“temporal weight”) obtained from the Ordinary Least Squares regression. The top panels show all absolute weights (“All β”). The bottom panels show the weights which were significantly different (“Significant β”) from zero (*p* < 0.05). There is a trend for the median absolute weightings in “Significant β” on the first click to be larger than for the other clicks, but the trend appears to be very modest when compared to the behavioral TWFs seen in Fig. 2, where the weights on the first click were an order of magnitude larger than those seen on the later clicks. The statistical significance of that trend is doubtful and very difficult to assess accurately given the non-normal nature and the nested statistical dependencies of the individual observations

To provide an overview of our complete dataset of 12 electrode placements across the 9 animals, we show boxplots in Fig. 6 which give the distributions of temporal weights (absolute regression coefficients |*β*|). We chose to plot absolute *β* values because the sign of the *β* depends on whether the recorded neural population at each electrode site happens to have a preference for ipsilateral or contralateral leading ITDs, and the sign is therefore not relevant to the question of whether the first or second pulse in the stimulus has a stronger influence on the amplitude of the response. Our data set comprises a total of 4 *β* coefficients (one for each pulse in the 4 pulse train) for each of the 61 electrode channels for each of the 12 electrode placements, yielding a total of 2928 coefficients at each of the two pulse rates tested. Of these, 147 (5%) were individually significant at alpha 0.05 at 300 Hz, and 228 (7%) were significant at 900 Hz. (Note that one would expect a priori that the proportion of significant regression weights obtained in this analysis is likely to be small). Boxplots summarizing the distributions of these *β* values are shown separately for the 300 and 900 Hz pulse rate conditions, and we show both the entire distribution of all regression weights and the distributions containing only weights which were significantly different from zero (*p* < 0.05).

The median absolute beta values for the first, second, third, and fourth pulses were similar in all conditions. For the distributions that excluded non-significant betas, there was a weak trend for median absolute weight of first, onset pulse to be larger than the other weights, but this trend is surprisingly weak compared to the robust behavioral onset weighting shown in Figs. 2 and 3). Note in Fig. 5 that TWFs seen at neighboring recording sites are often highly correlated. Therefore, regression weights obtained from neighboring sites cannot be treated as statistically independent observations, which makes it very difficult to judge whether the observed trends in the ECoG-derived weight distributions are statistically significant. In any event, the “effect size” of the weak trend seen in Fig. 6 appears too small, and the neural response patterns are too variable to provide a convincing physiological correlate of the very strong and consistent onset weighting seen in the behavioral results.

### Multivariate decoding shows strong precedence effect in ECoG signals from the auditory cortex

**Fig. 7:**
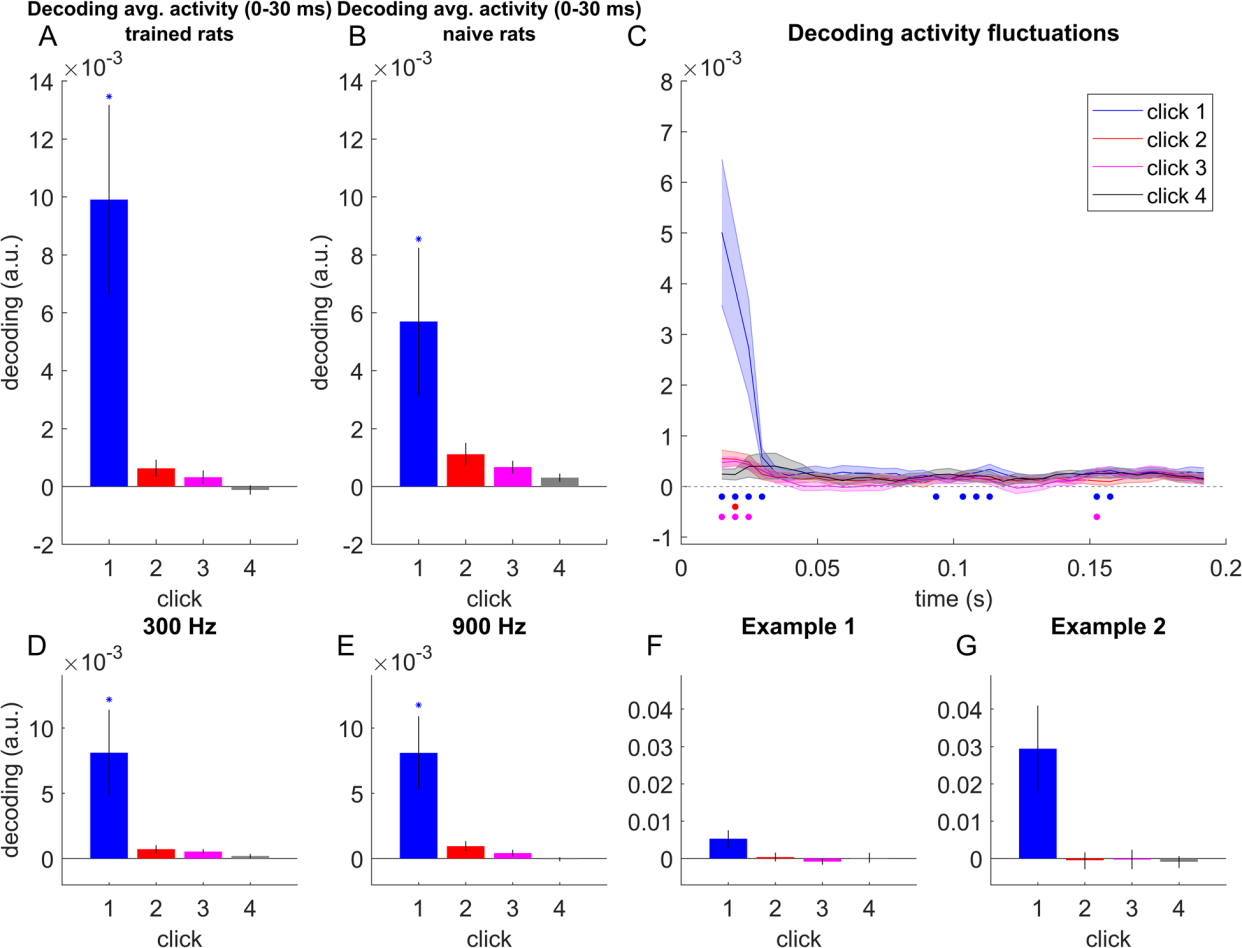
Multivariate analysis of auditory cortex ECoG responses. **A, B** ITD decoding for trained (seven placements) and naive (five placements) rats, respectively, based on average RMS activity between 0 and 30 ms relative to stimulus onset. Error bars denote SEMs across ECoG array placements. Asterisk indicates statistical significance (*p* < 0.05, Bonferroni-corrected). **C** ITD decoding time series based on activity fluctuations in each 30-ms-long sliding time window. Shaded areas denote SEMs across twelve placements. Filled circles indicate statistical significance (*p* < 0.01, FDR-corrected). **D, E** ITD decoding based on average RMS activity between 0 and 30 ms relative to stimulus onset, separately for 300 and 900 Hz pulse rates. Error bars denote SEMs across twelve ECoG array placements. Asterisks indicate statistical significance (*p* < 0.05, uncorrected). **F, G** Examples of ITD decoding based on average RMS activity between 0 and 30 ms relative to stimulus onset, separately for two individual electrode placements. Example 1 corresponds to Fig. 5A; example 2 corresponds to Fig. 5B. Error bars denote 99% confidence intervals

We have just seen that the univariate (channel-by-channel) analysis revealed only a weak trend toward onset weighting in the physiological TWFs obtained by regression analysis, and many individual recording sites showed the strongest weighting for pulses other than the first. In order to try to account for this apparent discrepancy, we investigated whether a multivariate stimulus-decoding analysis, which takes into account distributed activity patterns across the electrode array, can reveal a neural representation of stimulus ITDs which more accurately reflects the powerful onset weighting seen in the behavioral data (see section “Multivariate analysis: population-based decoding” for details). Indeed, this population decoding approach revealed very strong and highly significant onset weightings in the physiological responses which closely mirrored the behavioral results. When analyzing the average RMS activity observed within the first 30 ms after pulse train onset, and pooling RMS values over multiple channels the ITD decoding estimate of the first pulse was much higher than that for the other pulses, as is shown in Fig. 7A for data recorded from our four trained rats, and in Fig. 7B for the five naive rats. The results from the trained and naive animals were similar. While the decoding estimate for the initial pulse was nominally larger for the trained rats (mean 0.0099, *SEM* 0.0037) than for the naive rats (mean 0.0057, *SEM* 0.0034), the difference was not significant (Wilcoxon rank sum test, *Z* = 1.4569, *p* = 0.14). The difference between decoding estimates based on left and right cortex was also not significant (Wilcoxon rank sum test, *Z* = 0.506, *p* = 0.61; left: mean 0.0065, *SEM* 0.0027; right: mean 0.0087, *SEM* 0.0034). When pooling data for the trained and naive rats together, we found the decoding of the first three pulses to yield values that were significantly different from zero (first pulse: *p* < 0.001, *Z* = 3.945; second pulse: *p* = 0.019, *Z* = 2.346; third pulse: *p* = 0.042, *Z* = 2.033), while the decoding of the fourth pulse was not significant (*p* = 0.5663). The decoding of the first pulse was significantly higher than the decoding of all remaining pulses (all *p* < 0.003), and the decoding of the second pulse was significantly higher than the decoding of the fourth pulse (*p* = 0.019), while there were no significant differences in decoding between the remaining pulses (all *p* > 0.05). The decoding of the first pulse was also significant when both pulse rates were analyzed separately (300 Hz: *p* = 0.009, *Z* = 2.599; 900 Hz: *p* = 0.003, *Z* = 2.934; Fig. 7D, E). Overall, based on neural responses to the pulse trains pooled from multiple ECoG channels, the ITD of the first pulse (and, to a lesser extent, of the subsequent two pulses) could be decoded reliably from the cortical population response. These multivariate decoding results thus closely parallel the behavioral results, which show that the rats base their sound direction judgments predominantly on the first pulse.

In a further analysis, we investigated the decoding dynamics in a longer time window, ranging from 0 to 200 ms post stimulus onset (Fig. 7C). In this analysis, rather than using time-averaged activity to decode ITD, we used a sliding time window approach, in which we decoded ITD based on RMS fluctuations within 30-ms-long time windows, with a time step of 5 ms. Decoding based on activity fluctuations (rather than average activity) makes this analysis especially sensitive to short-lived transients in neural activity [19]. This analysis revealed that the ITD of the first pulse pair could be decoded based on early neural responses, corresponding to time windows centered at 15–30 ms following stimulus onset (*p*_FDR_ < 0.01, corrected across time windows), but also based on later neural responses (95–115 ms and 155–160 ms following stimulus onset; *p*_FDR_ < 0.01). Similarly to the decoding analysis based on average activity, in this analysis, we also observed weaker but significant decoding of the ITDs of the second and third clicks (click 2: 20 ms; click 3: 15–25 ms and 155 ms following pulse train onset; all *p*_FDR_ < 0.01, corrected across time windows; click 4: no significant decoding time windows), suggesting that brief neural transients to individual clicks might be sensitive to individual ITDs at different latencies. However, in this analysis too, the peak ITD decoding value for the first click was significantly (all *p* < 0.001) larger than those for the later clicks, by approximately an order of magnitude. The decoding results in Fig. 7C thus closely mirror the behavioral results shown in Fig. 2.

In Fig. 7F, G, we plot the individual decoding estimates for the same two examples as plotted in Fig. 5A, B, respectively. For both examples, the decoding of the first click ITD is better than that of the remaining clicks. Perhaps surprisingly, this trend is even more pronounced for the electrode placement where individual channels were heterogeneous and showed no obvious preference for the first click in the univariate analysis (example 2; Fig. 5B) than for the electrode placement in which univariate TWFs often showed a similar onset preference across the majority of channels (example 1; Fig. 5A). This is likely due to the fact that we used a Mahalanobis distance metric to estimate ITD decoding, in which the influence of each channel is scaled by the overall covariance across channels. Therefore, in the case of example 1, where most channels show a similar TWF pattern, the decoding relies on fewer response features than in the case of example 2, where different channels can have a unique contribution to decoding.

## Discussion

### Principal findings

To the best of our knowledge, this is the first study to measure binaural TWFs behaviorally and physiologically in a species other than humans. We were able to show that laboratory rats strongly onset weight binaural cues, basing lateralization judgments almost entirely on the first pulse in a click train. Their perception of such stimuli thus closely resembles that reported for human listeners. Note also that prior human psychoacoustic studies reported somewhat smaller weights on the first pulse when pulse rates were fairly low (20 or 100 Hz, inter-click intervals (ICI) of 50–10 ms) compared to those obtained with faster rates (500 Hz; ICIs of 2 ms) [8]. Our rat data exhibit the same trend, with the weight of the first pulse in a train increasing at higher pulse rates. We acknowledge that the number of animals in the behavior cohort was quite small (*N* = 4) but given that the behavioral results were highly consistent across all animals in that cohort, as well as with the multivariate analysis of our electrophysiological results from a larger cohort (*N* = 9), and with previously published human data, showing the striking similarities in TWF shapes and pulse-rate-related trends, our results nevertheless indicate that the heavily onset-weighted TWFs one would expect given the well-known precedence effect are likely to be the rule, rather than the exception, in laboratory rats.

Having thus validated the rat as a good model for human binaural hearing, we were able to follow this up with a physiological examination of the encoding of binaural cue values in cortical population responses recorded with ECoG arrays. Perhaps surprisingly, neural responses obtained at individual recording sites often failed to show a robust onset dominance in the temporal weighting of ITDs. However, a multivariate analysis of single-trial population responses revealed a neural encoding of ITDs that mirrored the animals’ behavioral responses. This observation has important implications for future studies, which may wish to use physiological tests on rats to probe factors governing binaural hearing performance, for example, in order to develop improved neuroprosthetic devices. Studies focusing heavily on reading out cortical responses one neuron or one unit or one recording site at a time could be missing out on important correlates of psychoacoustic performance that are only readily apparent at the level of a population coding analysis.

Only female rats were used in this study, as it is in our experience easier to run a behavior lab comprising only of female rats, avoiding “distractions” that would otherwise arise from pheromones and reproductive drives in our experimental animals. In mammals, both sexes derive very similar evolutionary benefits from spatial hearing and have a generally very similar auditory physiology and anatomy. We are aware of only single study of spatial hearing in humans which recruited the very large cohorts needed to measure the very small sex differences one might see, and they reported a very modest, but significant, difference, with females having ITD thresholds that are on average 0.25 sigma worse than those of males [20]. Sex differences of such small magnitude are negligible in the context of our research question, whether rats have essentially similar TWFs as humans. Given the internally very highly consistent results of our behavioral experiments, as well as the external consistency with the wealth of prior papers published on TWFs in humans and the precedence effect in general in a number of mammalian species, we do believe that our conclusion that rats show TWFs which are fundamentally similar to those seen in humans and other species is well supported.

### Neural correlates of the precedence effect

As discussed in [21], although the basic features of the precedence effect have been described for more than half a century, its biological mechanisms remain controversial. Electrophysiological recordings from central auditory structures have demonstrated reduced neural responses to “simulated echoes,” that is, pairs of pulses, similar to the first two pulses in the click trains used in this study [22–24]. It is natural to assume that a reduction in strength of auditory midbrain responses to the second click in a pair might lead to a corresponding down-weighting of that second click “further downstream,” as the neural representations of the pulses are fused into a unified percept of a single source with a (usually not consciously perceived) echo, and with a single perceived source location. Rather than challenging this assumption, much previous work has instead focused on discussing the physiological mechanisms that are likely to be responsible for the reduction of the response to the second click. The two main mechanisms considered were synaptic inhibition [25–27] or more peripheral processes involving cochlear mechanics [28, 29]. Central inhibition and cochlear mechanics could, in principle, both contribute to the physiological mechanisms which underpin the precedence effect. However, recent evidence suggests that cochlear mechanics are unlikely to be the major determinant, given that focal lesions [30] and pharmacological manipulations [25, 26] of auditory midbrain structures which leave the cochlea untouched nevertheless alter the precedence effect, and given that psychoacoustic signatures of a precedence effect have been observed even in cochlear implant patients in whom cochlear mechanics have been completely bypassed by neuroprosthetic stimulation [21].

Our approach to studying possible physiological bases for the precedence effect differs from previous work in a number of important ways. For example, previous physiological studies commonly measured responses in the inferior colliculus to each of two click pairs delivered in rapid succession in the inferior colliculus and interpreted an observed suppression of the lagging click as a putative correlate of the precedence effect. However, potential echos can arrive very quickly if reflective surfaces are slow. The highest pulse rate tested with our TWF paradigm is 900 Hz, corresponding to inter-pulse intervals of 1.1 ms, which is much too fast for the IC to produce individual responses locked to individual clicks [31]. Therefore, here we asked how strongly the neural response as a whole is influenced by the ITD of a given click (univariate, channel-wise regression analysis), and to what extent the response might be “decoded” to reveal the ITD of each click (multivariate analysis). Also, our study looks at neural responses in the cortex rather than IC. In this context, it is worth mentioning that a previous non-invasive study on human volunteers had described parallels between location judgments under conditions that required echo suppression and auditory evoked potentials [32].

Our investigation of the neural correlates of TWFs using channel-wise regression of individual channels produced very variable and inconsistent results, with some recording sites showing the expected largest weights for the first click in the train, but many others showing highly unexpected TWF patterns, with the largest absolute weight placed on the 2nd or 3rd pulse, in the middle of the 4-pulse train (cf Fig. 5C, D). The frequent, large, and significant weightings of later clicks is highly surprising because it implies that, at the level of individual groups of cortical neurons, neural TWFs can differ substantially from the behavioral TWF and place the largest weight away from the stimulus onset. This phenomenon was so common that, overall, there was only a small trend for the first pulse in the series to have the largest median absolute weight. This surprising finding led us to hypothesize that, at the level auditory cortex, a consistent precedence effect may only emerge at the level of neural population responses. To test this hypothesis, we analyzed the ECoG data using recently developed neural population decoding methods. That approach produced results in perfect agreement with the precedence effect demonstrated in the behavioral tests.

The two analysis approaches are conceptually and mathematically distinct, and the observed discrepancies between the two sets of results might be due to several reasons. One plausible explanation is that decoding the activity of a wider population of neurons is needed to observe the precedence effect. The regression analysis was applied here to analyze neural activity on a channel-by-channel basis, with a more localized, smaller set of neurons contributing to the responses within one channel. Conversely, the multivariate decoding method selected channels with a high signal-to-noise ratio, and pooled signals from these channels, integrating activity patterns over a much larger population of neurons. In the multivariate analysis, we also scaled the responses of each channel by their noise covariance, accounting for dependencies between channels. The success of the multivariate pattern analysis that we used for neural decoding suggests the following insights into the neural correlates of the precedence effect: First, pooling neural activity over space (channels)—which can enhance neural decoding accuracy [33, 34]—appears to be necessary to uncover a compelling neural correlate of the precedence effect. Second, pooling neural activity over time (i.e., including temporal transients rather than time-averaged responses as decoding features)—which highlights dynamic, short-lived neural activity patterns [19]—uncovered weaker but significant decoding of the later clicks in the train. Therefore, although the precedence effect strongly dominates the neural population code revealed by this analysis, read-outs of this code at fine temporal resolution nevertheless give access to binaural cue values beyond the very onset of the stimulus. This temporal integration of the signals to combine information from separate populations is also reminiscent of observations by [35] that the onset and offset of sound stimuli may be represented by non-overlapping populations of primary auditory cortex neurons (A1).

### The role of auditory cortex in spatial hearing and the precedence effect

Despite decades of study, there are still many unknowns about the role of auditory cortex in encoding spatial location in general, ITD cues in particular, and almost nothing is known the extent to which putative cortical encodings of space are subject to the precedence effect. Here our coverage of the cortical ITD literature is selective to focus on the those studies most relevant to our findings. That neurons in auditory cortex, including in high-frequency areas, can be tuned to ITDs, sometimes very sharply, has been documented [36], and the idea that some form of neural population code is likely involved in the cortical representation of auditory space is also not new [37]. Our neural decoding results extend these observations by showing a strong precedence effect in the AC of rats for click train stimuli at both 300 and 900 Hz, which nicely paralleled the behavior results. Nevertheless, this correspondence is of course not sufficient to prove that the precedence effect results from cortical population coding. Indeed, despite decades of study, the exact role of AC in spatial hearing remains somewhat unclear, and there may be substantial species differences. Unilateral lesions of AC result in poor performance in localizing brief sound in the contralateral sound field in a variety of species [38–41], while other studies indicate an important role for A1 in recalibrating binaural hearing after periods of partial monaural deprivation [42]. However, unilateral lesions on the contralateral AC of rats reportedly did not disrupt the sound localization performance of rats [43]. Also, how difficulties in localizing sources within one hemisphere relate to the sound lateralization ability across the midline has not been investigated in detail. There are also previous studies documenting ITD sensitivity at the level of AC of rats [44, 45], as well as studies on humans and cats which suggest that an intact AC may be necessary for the precedence effect [4, 46]. The fact that our ECoG data reveal fairly widespread, significant ITD sensitivity in LFP responses is therefore unsurprising. However, our finding that population decoding is necessary to reveal a strong precedence effect which mirrors behavioral observations is surprising and suggests an unexpected key role that cortex may play in transforming physical binaural cue values into integrated percepts.

## Conclusions

We developed a behavioral temporal weighting test for rats, based on previous human work [6]. The behavioral ITD TWFs of rats closely resemble those of humans, with the first (onset) pulse in a pulse train dominating the lateralization percept, and this onset weighting is particularly pronounced at faster pulse rates. The ECoG signals from AC of rats showed a population neural responses correspondence to behavioral TWFs, while the responses from a single recording site failed to reflect the behavioral TWFs most of the time. Our behavioral and electrophysiological findings confirmed that the precedence effect is preserved in rats. And the precedence effect is presented only in population neurons which may inspire the studies on “higher-order” perceptions to see questions in a greater map.

## Methods

### Animals

Our subjects were nine female Wistar rats which were 8-week-old and weighed 216–242 g at the beginning of the study. Of these, four were first used for behavioral training and psychoacoustic determination of ITD TWFs (see “Behavioral study” section). All nine (4 trained and 5 naive animals) were then used in terminal electrophysiological experiments to elucidate the cortical encoding of the binaural stimuli (see “Electrophysiology” section). The rats were housed in standard cages with 2 or 3 rats in each.

Preyer’s reflexes were tested, and the outer ears and tympanic membranes were visually examined to ensure that the rats had healthy, sensitive hearing. In addition, prior to the behavioral and electrophysiological experiments, acoustic brainstem responses (ABRs) were recorded to confirm normal hearing sensitivity (data not shown). For this examination, the rats were anesthetized by intraperitoneal injection of ketamine (80 mg/kg, 10%, Alfasan International B.V., Holland) and xylazine (12 mg/kg, 2%, Alfasan International B.V., Holland). Eye gel (Lubrithal, Dechra Veterinary Product A/S Mekuvej 9 DK-7171 Uldum) was applied to prevent the eyes from drying. The outer ear canals and tympanic membranes were inspected under a microscope (RWD Life Sciences, China). The rats were then fitted to a stereotactic instrument with a pair of hollow ear bars in a sound attenuating chamber, and ABRs to pulses were recorded to ascertain low hearing thresholds in both ears. ABR thresholds less than 30 dB SPL were considered indicative of normal auditory sensitivity. All the rats we used here had ABR thresholds < 30 dB. More detailed description of ABR recordings can be found in [15].

### Behavioral study

#### Behavioral training setup

The behavioral setup and the training methods were identical to those described in sections “Channel-wise regression shows only sporadic precedence effects in electrocorticographic (ECoG) signals from the auditory cortex” and “Multivariate decoding shows strong precedence effect in ECoG signals from the auditory cortex” of [14]. In brief, a training box was situated in a sound attenuating box and the front wall of the training box was fitted with three brass water spouts. Two hollow tubes were connected to a pair of mini headphone drivers (GQ-30783-000, Knowles, Itasca, Illinois, USA) to deliver the sound stimuli delivered by a USB sound card (StarTech.com, Ontario Canada, part No. ICUSBAUDIOMH) and amplified by an audio amplifier (Adafruit stereo 3.7 W class D audio amplifier, part No. 987) into the behavioral training box as close to the rats’ ears as possible. Stimulus delivery and monitoring and control of the behavioral task were performed by a Raspberry Pi computer running custom written Python software.

#### Behavioral training task

During behavioral training and testing, the four animals were tested five days a week, with two rest days. Our training used drinking water as a positive reinforcer. Therefore, a day prior to the first testing day, the home cage water bottles were removed, and for the following 5 days, the rats only had access to drinking water during their twice daily testing sessions. They then had easy access to ad lib water from the evening of the fifth training day until the morning of the second rest day. Food was available ad lib in the animals’ home cages throughout.

Behavioral training and testing were essentially identical to [14], except that a different set of stimuli was designed and used to enable the quantification of TWFs. In the behavioral experiments, rats performed a 2-AFC near-field lateralization task. Rats initialized each trial by licking a centrally positioned “start spout.” Initiating a trial was rewarded with a small drop of water on a random subset of 1 in every 7 trials. Initiating a trial triggered the delivery of a binaural stimulus, to which the animals responded by licking one of two “response spouts” positioned either side of the start spout. If the animal’s choice corresponded to the side indicated by the binaural cues, it was rewarded by three small drops of water delivered through the response spout. If the response was incorrect, it triggered a 15-s “timeout” during which a 90-dB negative feedback sound was played and no new trials could be initiated. If the rat made a wrong response, the following trial would be a “correction trial,” in which the last stimulus was repeated. “Correction trials” help reduce the tendency of animals to develop responses biases toward one side, but are excluded from the calculation of the correct response scores. Each rat performed two sessions per testing day, one in the morning and one in the afternoon, each session lasting ~ 20 min. The animals would typically perform between 100 and 200 trials per session.

The rats were initially trained with 200-ms-long, 300 Hz binaural pulse train stimuli which contained both ILD (± 6 dB) and ITD cues (± 0.136 ms). The motivation for the initial combined ITD and ILD training was to start the animals off with stimuli that should be very easy to lateralize as they contain “natural” combinations of both ITD and ILD binaural cues with rats to quickly adapt to the training environment. We required that the rats lateralized these initial training stimuli at least 80% correct in at least 2 sessions to advance to the next “ITD-only” training stage, during which ILDs were set to 0 dB. The rats reached the 80% correct criterion in this first stage of training after 8–10 days of training. After the initial training phase, all stimuli presented throughout the rest of the study had 0 dB ILDs and varied in ITD only. Once the rats reached 80% correct on two sessions with the 0.136-ms ITD-only stimuli, we increased the range of ITD values tested in each session. During this stage, the ITDs presented at each trial were drawn at random from the set ±[0.1587, 0.136, 0.0907, 0.068, 0.0454, 0.0227] ms. This set purposefully includes some ITD values that are below previously determined perceptual ITD thresholds for rats (~ 0.05 ms, see [14]), in order to accustom the animals to the possibility that sessions may include trials that may be difficult to lateralize. In this potentially more difficult “wide ITD range” training stage, the rats had to reach 75% correct in at least two sessions before advancing to the TWF testing stage. Animals which did not quickly advance to that final stage were given additional training sessions with easier stimuli, during which timeouts and reward quantities were individually adjusted as necessary to achieve reliably high levels of performance.

#### Acoustic stimuli for behavioral TWF measurement

The stimuli used in the TWF measurement phases of our experiments were modeled on the stimuli developed by [6]. Our stimuli consisted of trains of 8 binaural pulses (Fig. 1) delivered at rates of either 20, 50, 300, or 900 Hz. Importantly, the ITD of each pulse in the train was varied by introducing a small, random “temporal jitter” in the timing of each pulse which was independent in each ear. An analysis of lateralization judgments for a large number of stimuli with different ITD values at each pulse in the series would then make it possible to determine the “weight” (that is, the contribution made) by the *n*th pulse in the train to the perceived lateral position of the pulse train as a whole.

A potential difficulty with the use of these stimuli is that one cannot always determine a priori whether a subject’s lateralization response is “objectively correct.” Because ITD cue values of different pulses in the train could, by design, point in opposite directions, and how such ambiguous stimuli with conflicting ITD values are “supposed to be perceived” depends on the subject’s own TWF which is unknown at the start of the experiment. Brown and Stecker [7] simply trusted their human participants to understand the objective of the experiment and to report their lateralization judgments faithfully, presumably with low error or bias, and without the need of trial-by-trial reinforcement. Our rats, in contrast, need to be kept motivated and honest throughout the experiment by regular rewards for “correct” responses. Therefore, we constituted each block of trials as a randomly interleaved set of “honesty trials” and “probe trials.” In honesty trials, the ITDs of all pulses in the 8-pulse sequence pointed in the same direction, that is, they were either all positive (right ear leading) or all negative (left ear leading), so that the response to these honesty trials could be judged objectively as correct or incorrect irrespective of the details of each animal’s TWF. Pulses in honesty trails had a fixed ITD offset of ± 0.083 ms, plus an additional jitter drawn uniformly at random from a range of ± 0.042 ms, in steps of 10.4 μs afforded by the 96 kHz sample rate Hi-Fi USB Audio sound card. Since most ITD values in an honesty trial should be above typical rat ITD thresholds reported in [14], we expected them to be relatively easy to lateralize correctly, and responses to honesty trials were only rewarded if the animal responded on the appropriate side. We required that the rats lateralized at least 80% of honesty trials correctly in at least two sessions before they would also receive “probe trials,” in which the ITD for each pulse was drawn independently and uniformly from the range of ± 0.125 ms and ITDs of subsequent pulses in the train were allowed to point in opposite directions. Responses to probe trials were always rewarded regardless of the side on which the animal responded. In each TWF testing session, honesty trials and probe trials were randomly interleaved at a ratio of 2:1. The large proportion of honesty trials ensured that random guessing without attending to the sounds was not an effective strategy for the animals and allowed us to monitor that the animals continued to report their lateralization percepts with good accuracy throughout. During informal testing, the authors were unable to distinguish honesty trials from probe trials just by listening to them, and there is no indication that the rats could distinguish these either. We therefore consider it safe to assume that the rats’ responses to the probe trials accurately reflected their lateralization judgments for these stimuli. After reaching the “ITD-only” lateralization training criteria described above, all four rats were also able to meet the 80% correct TWF honesty trial criterion after minimal training, as might be expected given that to casual human observers, TWF stimuli with jittered ITDs and stimuli with fixed ITD sound indistinguishable.

#### Behavioral data analysis

TWFs were computed from the responses to the probe trials only, and separately for each of the four pulse rates (20, 50, 300, and 900 Hz), by computing a Probit regression to fit the probability of a “right spout” response against the ITD values for each of the 8 pulse pairs in the train, using the open source Python function *statsmodels.discrete.discrete_model.Probit* [47]. The Probit regression model takes the form:

1 $$ y=\varPhi \left({x}^T\;\beta \right) $$

Here, *y* is the probability that the animal will respond on the right, *x* is the vector of the eight ITD values of the pulses in the train plus an added 1 for the intercept, ^*T*^ is the transpose operator, and *β* is the vector of coefficients, or “weights” attributed to each of the pulses, which are estimated by maximum likelihood. *Φ* is the cumulative Gaussian normal distribution. The fitted model thus assumes additive effects of the weighted ITD of each pulse in the train, and the set of coefficients *β* represent the animal’s TWF. Here we use the terms Probit coefficient and temporal weight interchangeably.

### Electrophysiology

#### ECoG recording apparatus

Acoustic stimuli were generated by RZ6 multi-I/O processor (Tucker-Davis Technologies, USA) and presented via a pair of custom-made speakers (AS02204MR-N50-R, PUI Audio, Inc.) fitted to the openings of the hollow stainless steel ear bars, which fixed the rat into a stereotactic instrument (RWD Life Sciences, China). The speakers were calibrated with a GRAS 46DP-1 microphone (GRAS Sound & Vibration A/S), and their transfer functions were compensated with an inverse filter to be flat over the range of 600 Hz to 20 kHz to ~ ± 3 dB.

Neural activity was recorded using a 61-channel ECoG array [48]. The flexible (~ 30 μm thin) ECoG array comprised 203-μm diameter circular electrodes arranged on an 8 × 8 square grid, with three of the four corner positions unoccupied, and a 406-μm spacing between neighboring electrodes. The array covered an area of 10.6 mm^2^.

The neural signal was captured through two Intan C3314 32 channel headstage amplifiers (Intan Technologies, USA) connected to a PZ5 neurodigitizer (Tucker-Davis Technologies, USA) and processed with an RZ2 bioamp processor (Tucker-Davis Technologies, USA). Python programs written by the authors were used to generate stimuli and save the recorded signals.

#### ECoG recording procedure

ECoG was recorded from the auditory cortex (AC). At first, rats were anesthetized as described in the ABR recording procedure in “Behavioral data revealed profound onset dominance”, and their scalp was shaved. Prior to ECoG recording, ABRs were tested again to make sure the ear bars were still in good position, followed by intraperitoneal injection of urethane (20%, 1 mL). If a toe pinch reaction was observed during the ECoG recording, an additional 1 mL of urethane was injected. The total amount of injected urethane was less than 7.5 mL/kg. Additionally, butorphanol (10 mg/mL, 0.2 mL/kg every 1–2 h, Richter Pharma AG, 4600 Wels, Austria) was subcutaneously injected for analgesia. A deep cut in the midline of the scalp was made, and the surgical field was exposed. Local anesthetic Lignocaine (0.3 mL, 20 mg/mL, Troy Laboratories Pty Ltd, Australia) was applied on top of the surgical area. A craniotomy was performed over the right, or, in most cases, both temporal cortices. From a point 2.5 mm posterior to Bregma, a line was drawn perpendicular to the sagittal suture to the temporal ridge, and the intersection of this line and the ridge was marked. The craniotomy area extended 5.0 mm posterior and 4.0 mm ventral from this intersection point, to allow the placement of an ECoG electrode array on the auditory cortex (primary auditory cortex, secondary auditory cortex dorsal area, secondary auditory cortex ventral area). A hole was drilled through the skull anterior to Bregma to fix a screw which served as a reference electrode to connect to the ground wire of the recording headstage amplifier.

After placing the ECoG electrode array on the AC, acoustic stimuli were presented to the rat. ECoG neural signals were recorded at 6 kHz sample rate. At the end of the recording experiments, the rats were euthanized with an overdose of Pentobarbital (1~2 mL, 20%, Alfasan International B.V., Holland).

#### ECoG data analysis

We attempted two approaches to analyze the electrophysiological responses, one “univariate” approach which used a regression model to try to account for neural response amplitudes observed at each individual recording site, and one “multivariate” approach which attempted to use recently developed population decoding analyses to reconstruct stimulus ITDs from single-trial population responses observed across the ECoG array. The multivariate approach turned out to be much more successful, which has interesting implications for the nature of the representation of perceived ITDs as we shall see below.

##### Univariate analysis: channel-wise regression

Our analysis of the responses recorded with these stimuli was based on the assumption that most ITD sensitive neurons in the central auditory pathway would be tuned so as to have a “preference” for ITDs pointing to the contralateral side, while a minority might have an ipsilateral preference, but very few should have tuning curves that are symmetric at either end of the ecological range of ITDs [49–51]. We further assumed that neural response amplitudes of contralaterally tuned units should consistently increase when contralateral leading ITDs are presented, irrespective of whether these contralateral ITDs occur at the first, second, or *n*th click. Similarly, for ipsilaterally tuned units, response amplitudes should consistently decrease when contralateral ITDs are presented. Under these simplifying assumptions, we can attempt to fit TWFs to the neural data using a simple multiple linear regression, which regresses response amplitude against the signs of the four ITDs in each stimulus.

This univariate analysis of ECoG voltage data was further based on standard methods for quantifying evoked response amplitudes from LFPs as follows. First, per channel, the signal was band-passed using a 4th order band-pass filter from 30 to 300 Hz (scipy.signal.butter(), scipy.signal.filtfilt()). This chosen frequency region covers the gamma to very-high-gamma frequency ranges which have previously been shown to correlate particularly highly with multi-unit responses of auditory cortical neurons [52]. The band-passed signal was downsampled by a factor of 4 to a sample rate of 1500 Hz (scipy.signal.decimate()) and the decimated multichannel data were denoised using the “denoising by spatial filtering” methods developed by [53]. The cleaned data were “re-referenced” by subtracting the median across all channels. Re-referencing to the median has been shown to make the re-referenced signal less susceptible to outliers [54]. Neural responses were then quantified by epoching the cleaned, re-referenced signals into data segments ranging from 1 to 30 ms post stimulus onset. This epoch was chosen by visual inspection to cover the onset response peak, and it is consistent with reports that the initial responses to acoustic stimuli in the rat auditory cortex peak approximately 20 ms after stimulus onset [55]. The epoched data were baseline-corrected by subtracting their mean [56], and the RMS amplitude was calculated for each response epoch. Outlier epochs with RMS amplitudes greater than three standard deviations above the median RMS amplitude were excluded from further analysis [57].

The distribution of RMS response amplitudes obtained in this manner was highly positively skewed. We log-transformed the RMS values to make them more suitable for linear regression analysis to obtain TWF values. Furthermore, we wanted to compute temporal weighting coefficients in normalized units which were insensitive to site-to-site or animal-to-animal variability in the range of observed voltage values that can result from variable electrode impedances or electrode placements. We therefore *z*-scored the log(RMS) values prior to regression analysis.

The transformed data were then subjected to an ordinary least squares (OLS) regression (statsmodels.api.OLS, [47]) with constant added (Eq. 2). The form of the regression model is

2 $$ y={x}^T\;\beta +\in $$

where *y* is the *z*-scored, log-transformed LFP amplitude observed in each trial, *x* is a vector of the regressors (the ITDs of the 4 clicks in ms, and the added constant to provide the intercept), *β* is the vector of regression coefficients (the neural TWF weights in units of standard deviations of log RMS LFP amplitudes per ms of ITD), and *ε* is an error term which, as usual for normal linear regression, is assumed to follow a Gaussian distribution. In addition to computing the regression weights *β*, the software returned *p* values indicating how likely it is that the corresponding *β* is significantly different from zero.

##### Multivariate analysis: population-based decoding

In addition to the mass-univariate (i.e., channel-by-channel) analyses described above, data were also subject to a multivariate analysis based on the response of the population of recorded neurons (i.e., pooling information from multiple channels). Rather than computing the “weight” of a given ITD in the train as a scaling factor that maps ITD values onto changes in response amplitude, the rationale of this analysis was to try to decode the ITD value of each click in the train on a trial-by-trial basis. This decoding analyzed the pattern of neural activity measured by multiple ECoG channels, and quantified the “weight” of each ITD in the stimulus train by how well the ITD value can be decoded from single-trial neural population responses. The decoding methods used were originally developed by [58, 59] as a means to analyze human EEG response data, and were recently adapted for the analysis of rat auditory cortex ECoG data [60]. To construct the decoder, we first selected channels that showed a robust evoked response to the click train. The criterion that we used for channel selection was based on the signal-to-noise ratio (SNR), defined for each channel as the ratio between the RMS of the signal in the first 30 ms after click train onset and the RMS of the signal in the last 30 ms prior to click train onset. Only channels with SNR > 3 dB were taken into the analysis. On average, 73.36% (SEM 8.22% across penetrations) met this criterion.

Following channel selection, for each ECoG array position, data from multiple channels were used to decode the ITD of each click in the train, one at a time, based on the RMS in the 0–30-ms time window following click train onset. To this end, we split the data into three sets: (1) the test trial itself, (2) the remaining trials with the same ITD value as the test trial, and (3) the remaining trials with a different ITD than the test trial. From these three sets, we obtained three vectors of average response RMS amplitude values concatenated across channels. We then calculated the multivariate Mahalanobis distance values between (1) the test trial vector and the average vector of trials with the same ITD, as well as (2) the test trial vector and the average vector of trials with a different ITD. The Mahalanobis distance values were scaled by the noise covariance matrix of all channels, i.e., the covariance based on single-trial residual RMS after removing the mean RMS from each trial [61]. The scaled Mahalanobis distance values, obtained for a given trial *k* relative to other “same” or “different” trials, were used to calculate the overall decoding distance metric according to the following equation:

3 $$ decoding(k)=\frac{distance\left(k, different\right)\hbox{-} distance\left(k, same\right)}{distance\left(k, different\right)+ distance\left(k, same\right)} $$

This procedure was carried out for each trial in turn in a leave-one-out cross-validation approach, and the resulting decoding values were averaged across trials to obtain ITD decoding estimates for each of the four clicks in the train. The decoding estimates were tested for statistical significance using a signed rank test. These signed rank tests were done for all electrode placements pooled together, as well as separately for 300 and 900 Hz click rates. The tests were corrected for multiple comparisons using Bonferroni correction. Furthermore, to plot representative examples of individual electrode placements, we calculated 99% confidence intervals of the observed individual decoding estimates by repeating the analysis over 1000 iterations, whereby in each iteration the ITD labels were randomly reshuffled, to obtain a surrogate distribution of decoding estimates.

We also explored whether neural activity later than the first 30 ms following click train onset can be used to decode click ITDs. To this end, we repeated the decoding analysis in a sliding time window approach, using a window length of 30 ms (with a time step of 5 ms). Specifically, for each time window, we extracted the RMS envelope (downsampled to 200 Hz to yield 7 RMS values per time window), de-meaned it by removing the average across the time window (separately for each channel), and concatenated the de-meaned values across channels [19]. The resulting vectors of RMS fluctuations in multiple channels were used to calculate the Mahalanobis distance metrics and the corresponding decoding estimates, as described above. Decoding time series were tested for statistical significance for each click pair and time point using a signed rank test, correcting for multiple comparisons using a false discovery rate of 0.01 [62]. Again, these statistical tests were applied for all electrode placements pooled together, as well as for 300 and 900 Hz click rates separately.

## Data Availability

All data generated or analysed during this study are included in this published article and supplementary information files. The datasets supporting the conclusions of this article are available in the https://gin.g-node.org repository, doi:10.12751/g-node.tdg4ly [63].

## Abbreviations

ABRs: Acoustic brainstem responses
AC: Auditory cortex
A1: Primary auditory cortex
CI: Cochlear implant
ECoG: Electrocorticographic
ITDs: Interaural time differences
ILDs: Interaural level differences
TWFs: Temporal weighting functions
2-AFC: Two-alternative forced-choice

## References

[1] Litovsky RY, Colburn HS, Yost WA, Guzman SJ (1999). The precedence effect. J Acoust Soc Am..

[2] Gardner MB (1968). Historical background of the Haas and-or precedence effect. J Acoust Soc Am..

[3] Hoeffding V, Harrison JM (1979). Auditory discrimination: role of time and intensity in the precedence effect. J Exp Anal Behav..

[4] Cranford JL, Oberholtzer M (1976). Role of neocortex in binaural hearing in the cat. II. The ‘precedence effect’ in sound localization. Brain Res..

[5] Stecker GC, Hafter ER (2002). Temporal weighting in sound localization. J Acoust Soc Am..

[6] Brown AD, Stecker GC (2010). Temporal weighting of interaural time and level differences in high-rate click trains. J Acoust Soc Am..

[7] Brown AD, Stecker GC (2011). Temporal weighting functions for interaural time and level differences. II. The effect of binaurally synchronous temporal jitter. J Acoust Soc Am..

[8] Stecker GC, Ostreicher JD, Brown AD (2013). Temporal weighting functions for interaural time and level differences. III. Temporal weighting for lateral position judgments. J Acoust Soc Am..

[9] Stecker GC (2014). Temporal weighting functions for interaural time and level differences. IV. Effects of carrier frequency. J Acoust Soc Am..

[10] van Hoesel RJM, Tyler RS (2003). Speech perception, localization, and lateralization with bilateral cochlear implants. J Acoust Soc Am..

[11] Laback B, Majdak P (2008). Binaural jitter improves interaural time-difference sensitivity of cochlear implantees at high pulse rates. Proc Natl Acad Sci..

[12] Goupell MJ, Laback B, Majdak P (2009). Enhancing sensitivity to interaural time differences at high modulation rates by introducing temporal jitter. J Acoust Soc Am..

[13] Litovsky R (2006). Failure of the precedence effect in children and in persons with hearing loss. J Acoust Soc Am..

[14] Li K, Chan CHK, Rajendran VG, Meng Q, Rosskothen-Kuhl N, Schnupp JWH (2019). Microsecond sensitivity to envelope interaural time differences in rats. J Acoust Soc Am..

[15] Rosskothen-Kuhl N, Buck AN, Li K, Schnupp JW (2021). Microsecond interaural time difference discrimination restored by cochlear implants after neonatal deafness. eLife..

[16] Koka K, Read HL, Tollin DJ (2008). The acoustical cues to sound location in the rat: measurements of directional transfer functions. J Acoust Soc Am..

[17] Stecker GC (2018). Temporal weighting functions for interaural time and level differences. V. Modulated noise carriers. J Acoust Soc Am..

[18] Fitzpatrick DC, Kuwada S, Batra R (2000). Neural sensitivity to interaural time differences: beyond the Jeffress model. J Neurosci..

[19] Wolff MJ, Kandemir G, Stokes MG, Akyürek EG (2020). Unimodal and bimodal access to sensory working memories by auditory and visual impulses. J Neurosci..

[20] Saberi K, Antonio JV, Petrosyan A (2004). A population study of the precedence effect. Hear Res..

[21] Brown AD, Jones HG, Kan A, Thakkar T, Stecker GC, Goupell MJ, et al. Evidence for a neural source of the precedence effect in sound localization. J Neurophysiol. 2015;114(5):2991–3001. 10.1152/jn.00243.2015. 10.1152/jn.00243.2015 PMC4737417 26400253

[22] Yin TC (1994). Physiological correlates of the precedence effect and summing localization in the inferior colliculus of the cat. J Neurosci..

[23] Litovsky RY, Yin TCT (1998). Physiological studies of the precedence effect in the inferior colliculus of the cat. II. Neural Mechanisms. J Neurophysiol.

[24] Tollin D, Populin L, Yin T (2004). Neural correlates of the precedence effect in the inferior colliculus of behaving cats. J Neurophysiol..

[25] Burger RM, Pollak GD (2001). Reversible inactivation of the dorsal nucleus of the lateral lemniscus reveals its role in the processing of multiple sound sources in the inferior colliculus of bats. J Neurosci..

[26] Pecka M, Zahn TP, Saunier-Rebori B, Siveke I, Felmy F, Wiegrebe L, et al. Inhibiting the inhibition: a neuronal network for sound localization in reverberant environments. J Neurosci. 2007;27(7):1782–90. 10.1523/JNEUROSCI.5335-06.2007. 10.1523/JNEUROSCI.5335-06.2007 PMC6673727 17301185

[27] Xia J, Brughera A, Colburn HS, Shinn-Cunningham B (2010). Physiological and psychophysical modeling of the precedence effect. J Assoc Res Otolaryngol..

[28] Hartung K, Trahiotis C (2001). Peripheral auditory processing and investigations of the “precedence effect” which utilize successive transient stimuli. J Acoust Soc Am..

[29] Bianchi F, Verhulst S, Dau T (2013). Experimental evidence for a cochlear source of the precedence effect. JARO..

[30] Litovsky RY, Fligor BJ, Tramo MJ (2002). Functional role of the human inferior colliculus in binaural hearing. Hear Res..

[31] Schnupp JWH, Garcia-Lazaro JA, Lesica NA (2015). Periodotopy in the gerbil inferior colliculus: local clustering rather than a gradient map. Front Neural Circuit..

[32] Liebenthal E, Pratt H (1999). Human auditory cortex electrophysiological correlates of the precedence effect: binaural echo lateralization suppression. J Acoust Soc Am..

[33] Grootswagers T, Wardle SG, Carlson TA (2017). Decoding dynamic brain patterns from evoked responses: a tutorial on multivariate pattern analysis applied to time series neuroimaging data. J Cogn Neurosci..

[34] Nemrodov D, Niemeier M, Patel A, Nestor A (2018). The neural dynamics of facial identity processing: insights from EEG-based pattern analysis and image reconstruction. eNeuro.

[35] Scholl B, Gao X, Wehr M (2010). Nonoverlapping sets of synapses drive on responses and off responses in auditory cortex. Neuron..

[36] Moshitch D, Nelken I (2016). The representation of interaural time differences in high-frequency auditory cortex. Cereb Cortex.

[37] Miller LM, Recanzone GH (2009). Populations of auditory cortical neurons can accurately encode acoustic space across stimulus intensity. Proc Natl Acad Sci..

[38] Cranford J, Ravizza R, Diamond IT, Whitfield IC (1971). Unilateral ablation of the auditory cortex in the cat impairs complex sound localization. Science..

[39] Jenkins WM, Merzenich MM (1984). Role of cat primary auditory cortex for sound-localization behavior. J Neurophysiol..

[40] Kavanagh GL, Kelly JB (1987). Contribution of auditory cortex to sound localization by the ferret (Mustela putorius). J Neurophysiol..

[41] Lomber SG, Malhotra S (2008). Double dissociation of 'what' and 'where' processing in auditory cortex. Nature neuroscience..

[42] Bajo VM, Nodal FR, Korn C, Constantinescu AO, Mann EO, Boyden ES 3rd, et al. Silencing cortical activity during sound-localization training impairs auditory perceptual learning. Nat Commun. 2019;10(1):3075. 10.1038/s41467-019-10770-4. 10.1038/s41467-019-10770-4 PMC6625986 31300665

[43] Kelly JB (1980). Effects of auditory cortical lesions on sound localization by the rat. J Neurophysiol..

[44] Kelly JB, Phillips DP (1991). Coding of interaural time differences of transients in auditory cortex of Rattus norvegicus: implications for the evolution of mammalian sound localization. Hear Res..

[45] Tsytsarev V, Fukuyama H, Pope D, Pumbo E, Kimura M (2009). Optical imaging of interaural time difference representation in rat auditory cortex. Front Neuroeng..

[46] Cornelisse LE, Kelly JB (1987). The effect of cerebrovascular accident on the ability to localize sounds under conditions of the precedence effect. Neuropsychologia..

[47] Seabold S, Perktold J (2010). Statsmodels: econometric and statistical modeling with python. Proceedings of the 9th Python in Science Conference.

[48] Woods V, Wang C, Bossi S, Insanally M, Trumpis M, Froemke R, et al. A low-cost, 61-channel μECoG array for use in rodents. In: 2015 7th International IEEE/EMBS Conference on Neural Engineering (NER). Montpellier: IEEE; 2015. p. 573–6. 10.1109/ner.2015.7146687.

[49] Benson DA, Teas DC (1976). Single unit study of binaural interaction in the auditory cortex of the chinchilla. Brain Res..

[50] Woldorff MG, Tempelmann C, Fell J, Tegeler C, Gaschler-Markefski B, Hinrichs H, et al. Lateralized auditory spatial perception and the contralaterality of cortical processing as studied with functional magnetic resonance imaging and magnetoencephalography. Hum Brain Mapp. 1999;7:49–66. 10.1002/(sici)1097-0193(1999)7:1<49::aid-hbm5>3.0.co;2-j. 10.1002/(SICI)1097-0193(1999)7:1<49::AID-HBM5>3.0.CO;2-J PMC6873307 9882090

[51] Yao JD, Bremen P, Middlebrooks JC (2013). Rat primary auditory cortex is tuned exclusively to the contralateral hemifield. J Neurophysiol..

[52] Steinschneider M, Fishman YI, Arezzo JC (2008). Spectrotemporal analysis of evoked and induced electroencephalographic responses in primary auditory cortex (A1) of the awake monkey. Cereb Cortex.

[53] de Cheveigné A, Simon JZ (2008). Denoising based on spatial filtering. J Neurosci Methods..

[54] Liu Y, Coon WG, de Pesters A, Brunner P, Schalk G (2015). The effects of spatial filtering and artifacts on electrocorticographic signals. J Neural Eng..

[55] Rutkowski RG, Miasnikov AA, Weinberger NM (2003). Characterisation of multiple physiological fields within the anatomical core of rat auditory cortex. Hear Res..

[56] Boatman-Reich D, Franaszczuk P, Korzeniewska A, Caffo B, Ritzl E, Colwell S, et al. Quantifying auditory event-related responses in multichannel human intracranial recordings. Front Comput Neurosci. 2010;4:4. 10.3389/fncom.2010.00004. 10.3389/fncom.2010.00004 PMC2859880 20428513

[57] Jas M, Engemann D, Raimondo F, Bekhti Y, Gramfort A. Automated rejection and repair of bad trials in MEG/EEG. In: 2016 International Workshop on Pattern Recognition in Neuroimaging (PRNI). Trento: IEEE; 2016. p. 1–4. 10.1109/PRNI.2016.7552336.

[58] Wolff MJ, Jochim J, Akyürek EG, Stokes MG (2017). Dynamic hidden states underlying working-memory-guided behavior. Nat Neurosci..

[59] Auksztulewicz R, Myers NE, Schnupp JW, Nobre AC (2019). Rhythmic temporal expectation boosts neural activity by increasing neural gain. J Neurosci..

[60] Cappotto D, Auksztulewicz R, Kang H, Poeppel D, Melloni L, Schnupp J (2021). Decoding the content of auditory sensory memory across species. Cerebral Cortex..

[61] Muhle-Karbe PS, Myers NE, Stokes MG. A hierarchy of functional states in working memory. J Neurosci. 2021;41(20):4461–75. 10.1523/JNEUROSCI.3104-20.2021. 10.1523/JNEUROSCI.3104-20.2021 PMC8152603 33888611

[62] Benjamini Y, Hochberg Y (1995). Controlling the false discovery rate: a practical and powerful approach to multiple testing. J R Stat Soc Ser B Methodol..

[63] Li K, Auksztulewicz R, Chan C, Mishra A, Schnupp J. The precedence effect in spatial hearing manifests in cortical neural population responses. G-Node. 2021. 10.12751/G-NODE.TDG4LY. 10.1186/s12915-022-01228-z PMC8848659 35172815

